# Biochemical Insights into Lipid Remodeling in Wheat Anthers Under High-Temperature Stress

**DOI:** 10.3390/ijms262311426

**Published:** 2025-11-26

**Authors:** Guang Chen, Peimin Zhao, Wenping Wang, Honghong Wu, Qiang Li

**Affiliations:** 1 National Key Laboratory of Crop Genetic Improvement, Huazhong Agricultural University, Wuhan 430070, China; 2020cheng@webmail.hzau.edu.cn (G.C.); zhaopeimin@webmail.hzau.edu.cn (P.Z.); wangwenping2023@163.com (W.W.); 2 The Center of Crop Nanobiotechnology, Huazhong Agricultural University, Wuhan 430070, China

**Keywords:** wheat, high-temperature stress, anther, pollen fertility, lipidome

## Abstract

High-temperature (HT) stress during flowering significantly impairs anther development and pollen fertility, leading to substantial yield loss in wheat. A key aspect of plant adaptation to temperature stress concerns remodeling of lipid metabolism. In this study, heat-tolerant and heat-sensitive wheat cultivars were employed to investigate the biochemical alterations in lipid metabolism in response to high-temperature (HT) stress during anthesis. Pollen viability and SEM demonstrated that, under high temperature, the heat-tolerant cultivar maintained a more stable pollen structure and exhibited higher pollen fertility compared to the sensitive cultivar. Fatty acid analysis showed that HT led to a decrease in the unsaturated fatty acid 18:3 and an increase in the saturated fatty acid 16:0, thereby reducing the double bond index in both cultivars. Lipidomic profiling revealed that HT caused a shift toward higher levels of saturated acyl chains, reducing unsaturation in both phospholipids and galactolipids. Notably, the levels of saturated lipids such as PC (34:0) and PA (36:0) increased markedly upon heat exposure in the heat-tolerant cultivar, whereas only minor changes were observed in the heat-sensitive cultivar. Furthermore, analysis of cuticular lipids showed a reduction in polyunsaturated cutin components under high temperature in wheat anthers. Heat treatment caused a substantial reduction in fertile spikelet rate in both cultivars, while the heat-tolerant cultivar maintained a better seed setting and higher yield. These findings provide biochemical insights into lipid metabolic adjustments that underlie thermotolerance during anthesis in wheat.

## 1. Introduction

Wheat (*Triticum aestivum* L.) is one of the most widely cultivated crops in the world, providing 20% of the calories and protein consumed by humans [1]. With climate change, adverse conditions affecting wheat production are becoming more frequent [2,3]. Climate change leads to wheat being exposed to warmer growing conditions, which can be detrimental to its growth and development once temperatures exceed the limit [4,5]. Wheat is sensitive to temperature throughout its various growth stages [6,7,8,9,10], with the reproductive phase being the most vulnerable [11,12]. During anthesis, pollen is particularly vulnerable when exposed to sub- or super-optimal temperature [13]. High temperatures during anthesis impair anther development as well as pollen morphology and function, resulting in reduced pollen fertility and ultimately, severe yield loss in wheat [14,15,16,17,18]. Therefore, elucidating the molecular mechanisms that confer heat tolerance is crucial for ensuring wheat productivity and global food security.

Lipids, including cuticular and membrane lipids, are essential components of plant cells and play critical roles in plant development and temperature adaptation [19,20,21,22,23,24]. A key adaptive mechanism involves the remodeling of lipid metabolism to maintain membrane integrity and fluidity under temperature stress, a process largely governed by the degree of acyl chain unsaturation in membrane lipids [25,26,27,28]. Adaptation to high-temperature stress is frequently associated with increased lipid saturation. For instance, a soybean mutant deficient in fatty acid desaturation exhibited strong thermotolerance [29]. In Arabidopsis, the *fab2* mutant, which is unable to convert 18:0-ACP to 18:1-ACP, accumulates saturated fatty acids and shows enhanced tolerance at 35 °C [30]. Similarly, two fatty acid desaturase mutants, *fad5* and *fad6*, which are deficient in 16:3 and dienoic/trienoic fatty acids, respectively, exhibit enhanced thermal stability under heat stress [31]. Moreover, *fad7* and *fad7*/*fad8* double mutants also demonstrate greater heat tolerance at 35 °C compared to wild-type plants [21]. In plants, lipid changes at different temperatures are not only observed in fatty acid composition and desaturation, but also in the proportion of glycerolipid species as well as their molecular compositions [32]. It has been shown that high temperature resulted in an increased production of phospholipids, while galactolipids biosynthesis was repressed [33]. Moreover, heat stress increases 18:3- and 16:3-containing phosphatidylcholine (PC) and diacylglycerol (DAG) species (e.g., 34:6-PC, 36:6-PC, 34:6-DAG, 36:6-DAG) in leaves [34,35]. In addition, a decrease in MGDG (18:3/16:3) relative to MGDG (18:3/18:3) was also observed in Arabidopsis grown at high temperatures [36]. Hence, lipid metabolic reprogramming represents a critical strategy in plant adaptation to temperature fluctuations.

Besides membrane lipids, cuticular lipids, including waxes and cutin polyesters, form a hydrophobic barrier on the surface of plant tissues. The cuticular layer plays essential roles in plant development, reproductive processes, and adaptation to environmental stresses [37,38,39,40]. Fatty acids and their derivatives are integral components of the anther cuticle and pollen exine. The pollen exine, primarily composed of sporopollenin derived from fatty acid precursors, protects the male gametophyte from environmental stress, mediates pollen-pistil interactions, and ensures successful fertilization and seed formation [41,42,43]. The anther cuticle, composed of waxes and cutin, serves as an extracellular lipid layer that shields reproductive tissues from abiotic stress, dehydration, and pathogen invasion [44]. A number of genes involved in lipid metabolism have been suggested to be involved in the development of the anther cuticle and pollen. In rice, cytochrome P450 family members catalyze fatty acid hydroxylation, a key step in sporopollenin biosynthesis, with mutations resulting in pollen sterility [45,46]. The biosynthesis of sporopollenin also affects the biosynthesis of cutin in the cuticle layer. Genes such as *OsGPAT3* in rice, *ZmMs33* in maize, *AtGPAT1* and *AtGPAT6* in Arabidopsis are involved in regulating the polymerization process of cutin monomers. Mutants lacking *ZmMs33* and *OsGPAT3* exhibit abnormal anther cuticles and poor development of pollen exine [47,48]. Despite the recognized importance of lipid metabolism in anther development and pollen fertility, its regulation under high-temperature conditions in wheat remains poorly understood.

In this study, we systematically analyzed lipid profiles in the anthers of heat-tolerant and heat-sensitive wheat in response to high-temperature stress. By examining the lipid metabolic changes and their pathway interactions during anthesis under heat stress, this study elucidates the role of lipid metabolism in thermotolerance, providing a theoretical foundation for breeding heat-resilient wheat varieties.

## 2. Results

### 2.1. High Temperature Impaired Pollen Fertility and Structure

**Figure 1:**
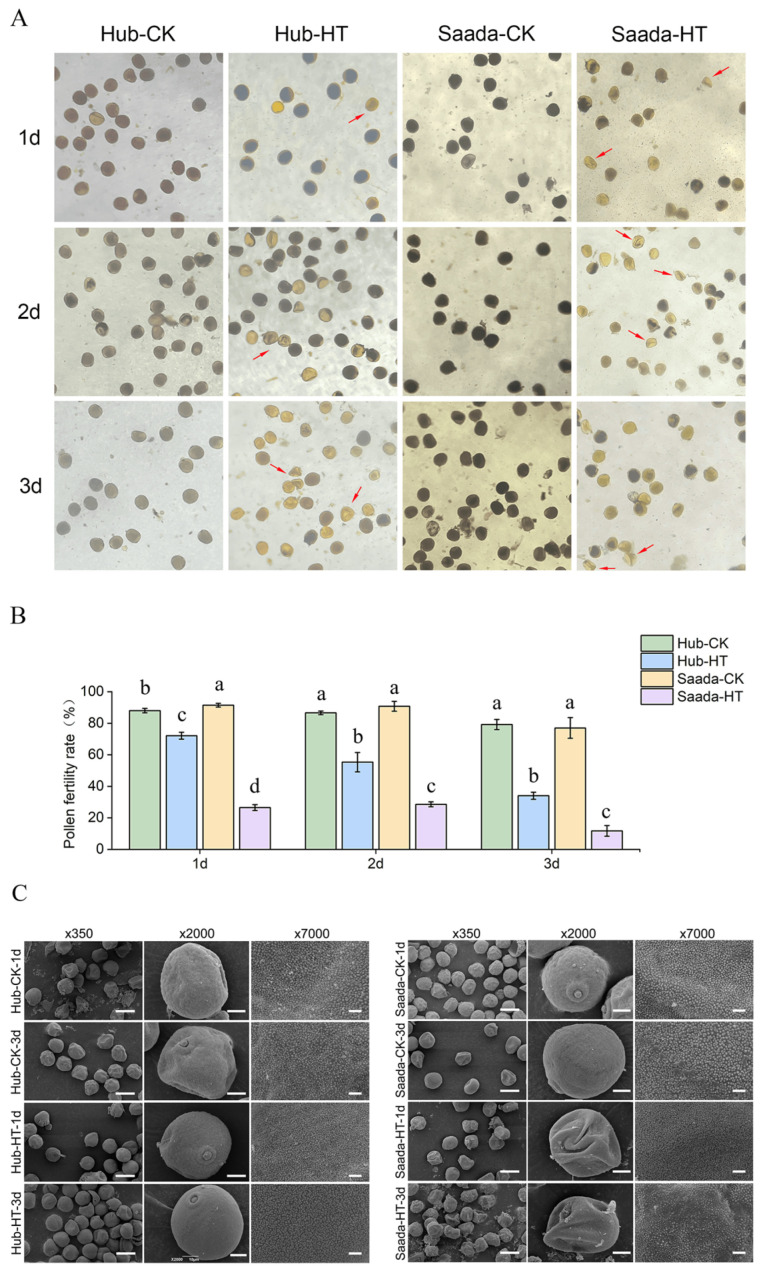
Effects of high-temperature stress on pollen viability and morphology. (**A**) Representative images of pollen grains stained with iodine-potassium iodide observed under a light microscope (10× magnification). Viable pollen grains were stained dark blue and aborted pollen grains stained yellow as indicated by the red arrows. (**B**) Pollen viability of heat-tolerant (Hub) and heat-sensitive (Saada) wheat lines under normal conditions (CK, 22 °C) and high-temperature (HT, 34 °C) treatment. Data are presented as mean ± SD, *n* = 3. Different lowercase letters indicate statistically significant differences among groups as determined by Fisher’s Least Significant Difference (LSD) test at *p* < 0.05. (**C**) Scanning electron microscopy (SEM) images of pollen grains from both lines under high-temperature stress, showing surface ultrastructure at increasing magnifications. Scale bars: 50 μm (×350), 10 μm (×2000), and 2 μm (×7000). HT, 34 °C; CK, 22 °C; 1d, the first day of high-temperature treatment; 2d, the second day of high-temperature treatment; 3d, the third day of high-temperature treatment.

Two wheat cultivars, a heat-resistant line (Hub), and a heat-sensitive line (Saada), were employed to investigate the physiological and biochemical mechanisms in response to high-temperature stress [49]. Both lines were grown under optimal conditions until heading, then exposed to 34 °C for three days once anthers extruded from the florets. Anthers were collected over three consecutive days during high-temperature treatment. Pollen developmental stages were determined by DAPI staining, which revealed that binucleate pollen was present on days 1 and 2, while trinucleate pollen was observed by day 3 in both cultivars (Figure S1). Pollen viability was assessed by iodine staining (Figure 1A). Under normal conditions, both lines exhibited fertility rates at approximately 90%. However, heat stress significantly reduced pollen viability in both cultivars (Figure 1B). The viability of the heat-sensitive cultivar (Saada) dropped sharply to 26.54% on the first day of high-temperature treatment and declined to 11.76% by the end of the treatment period. In contrast, the pollen viability of the heat-tolerant line (Hub) declined gradually, maintaining 72.15%, 55.33%, and 34.02% viability on the first, second, and third days of heat treatment, respectively. These results indicate that the pollen of the heat-tolerant cultivar exhibits greater resilience to high-temperature stress compared to the heat-sensitive line.

Pollen ultrastructure of the two cultivars was further examined by scanning electron microscopy (SEM) to assess morphological differences. Under normal conditions, both lines displayed similarly mild surface wrinkling and granular texture. Under high-temperature stress, the ultrastructure of Hub pollen remained intact, while Saada pollen showed pronounced surface wrinkling and a notable reduction in surface granules (Figure 1C). The structural defects in Saada, which paralleled its drastic loss of viability, implies that surface morphology deterioration may contribute to fertility reduction under heat stress.

### 2.2. High Temperature Reduced the Level of Fatty Acid Unsaturation in Anthers

**Figure 2:**
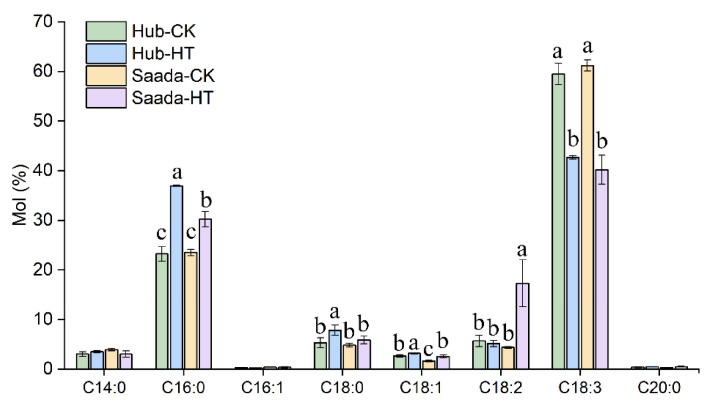
Fatty acid composition of wheat anthers under high-temperature stress. Anthers were sampled on the third day of high-temperature treatment (HT, 34 °C) and control (CK, 22 °C). Fatty acids are abbreviated as follows: C14:0, myristic acid; C16:0, palmitic acid; C16:1, palmitoleic acid; C18:0, stearic acid; C18:1, oleic acid; C18:2, linoleic acid; C18:3, linolenic acid; C20:0, arachidic acid. Data are presented as mean ± SD (*n* = 3). Different lowercase letters above bars indicate significant differences between cultivars within each fatty acid as determined by Fisher’s Least Significant Difference (LSD) test at *p* < 0.05.

Given the key role of lipid metabolism in pollen development and thermotolerance [50,51], we analyzed the fatty acid composition in both wheat lines under high-temperature stress. The major fatty acids were C16:0, C18:0, C18:1, C18:2, and C18:3. While no significant differences were found between the heat-tolerant Hub and heat-sensitive Saada under control conditions, both cultivars showed increased saturated fatty acids (C16:0, C18:0) and decreased polyunsaturated C18:3 under heat stress. Notably, the increase in saturated fatty acids was more pronounced in the heat-tolerant Hub. Conversely, a significant rise in C18:2 was observed specifically in the heat-sensitive Saada under stress, a change absent in Hub (Figure 2).

The double bond index (DBI) was calculated to evaluate membrane unsaturation. Under high-temperature treatment, a significant decrease in DBI was observed in both wheat lines. Notably, the heat-tolerant Hub exhibited a greater decline in DBI than Saada (Table 1), consistent with a more substantial shift toward a more saturated lipid composition in response to heat stress.

### 2.3. Effect of High Temperature on Anthers Lipidome

**Figure 3:**
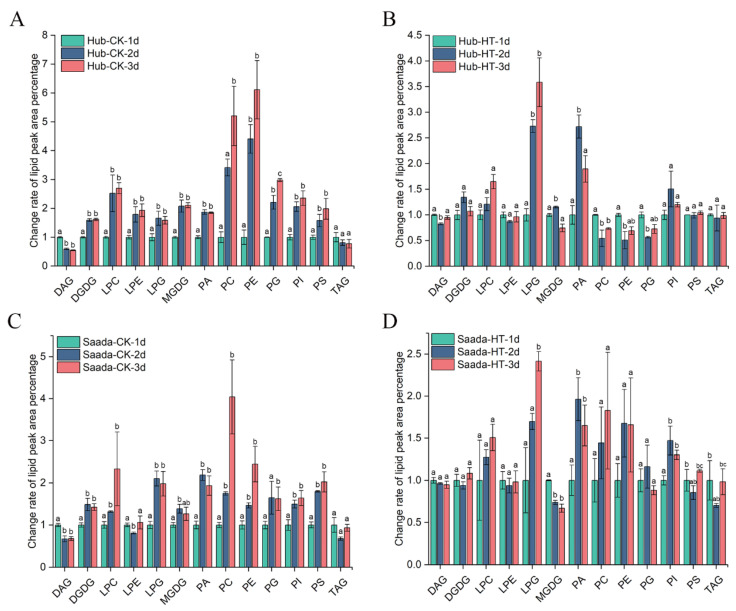
Lipidomics profiling of wheat anthers under high-temperature stress. The relative abundance of major lipid classes across the three days of flowering during normal growth (**A**,**C**) and heat treatment (**B**,**D**) in Hub and Saad, respectively. The *Y*-axis shows the relative levels calculated as the ratio of the peak area percentage for each lipid class on day 2 or 3 to its corresponding value on day 1. Lipids were extracted from 20 anthers per sample, with data representing mean ± SD from three biological replicates (*n* = 3). Different lowercase letters indicate significant temporal changes for each lipid class across days as determined by Fisher’s Least Significant Difference (LSD) test at *p* < 0.05. HT, 34 °C; CK, 22 °C; 1d, the first day of high-temperature treatment; 2d, the second day of high-temperature treatment; 3d, the third day of high-temperature treatment. DAG, diacylglycerols; DGDG, digalactosyldiacylglycerol; LPC, lysophosphatidylcholine; LPE, lysophosphatidylethanolamine; LPG, lysophosphatidylglycerol; MGDG, monogalactosyldiacylglycerol; PA, phosphatidic acid; PC, phosphatidylcholine; PE, phosphatidylethanolamine; PG, phosphatidylglycerol; PI, phosphatidylinositol; PS, phosphatidylserine; TAG, triacylglycerols.

The marked changes in fatty acid composition indicated significant alterations in lipid profiles [25]. To investigate the biochemical mechanisms underlying fatty acid changes, we performed lipidomics analysis to examine associated metabolic alterations during anther development under heat stress. A total of 364 lipid species were identified in wheat anthers, including triacylglycerols (TAG), diacylglycerols (DAG), phospholipids, and galactolipids. Under control conditions, our results revealed an increase in most phospholipids and galactolipids, concomitant with a decline in DAG (Figure 3A,C). These results suggested an active lipid biosynthesis and a conversion of DAG to glycerolipids during anther and pollen development. Under high temperature, the proportion of lysophosphatidylglycerol (LPG) and phosphatidic acid (PA) were increased significantly in both Hub and Saada, while the proportions of other lipid species remained relatively stable. In addition, the proportion of phospholipids such as phosphatidylcholine (PC), phosphatidylethanolamine (PE), and phosphatidylglycerol (PG) was significantly decreased in Hub (Figure 3B,D).

**Figure 4:**
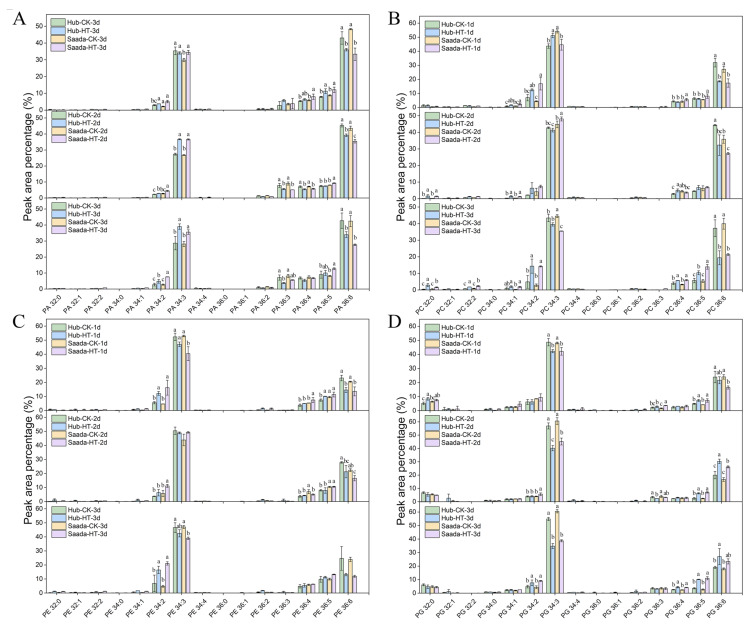
Compositional changes of phospholipids in wheat anthers under high-temperature stress. (**A**–**D**) The relative abundance of individual molecular species presented as a percentage of total peak area for phosphatidic acid (PA) (**A**), phosphatidylcholine (PC) (**B**), phosphatidylethanolamine (PE) (**C**), and phosphatidylglycerol (PG) (**D**). Anthers were sampled on the first three days (1d–3d) of flowering under normal conditions (CK, 22 °C) and high-temperature (HT, 34 °C) treatment. The *X*-axis denotes lipid molecular species (total acyl carbons: total double bonds). Data represent mean ± SD from three biological replicates (*n* = 3). For each specific lipid species, different lowercase letters indicate significant temporal changes across days as determined by Fisher’s Least Significant Difference (LSD) test at *p* < 0.05.

**Figure 5:**
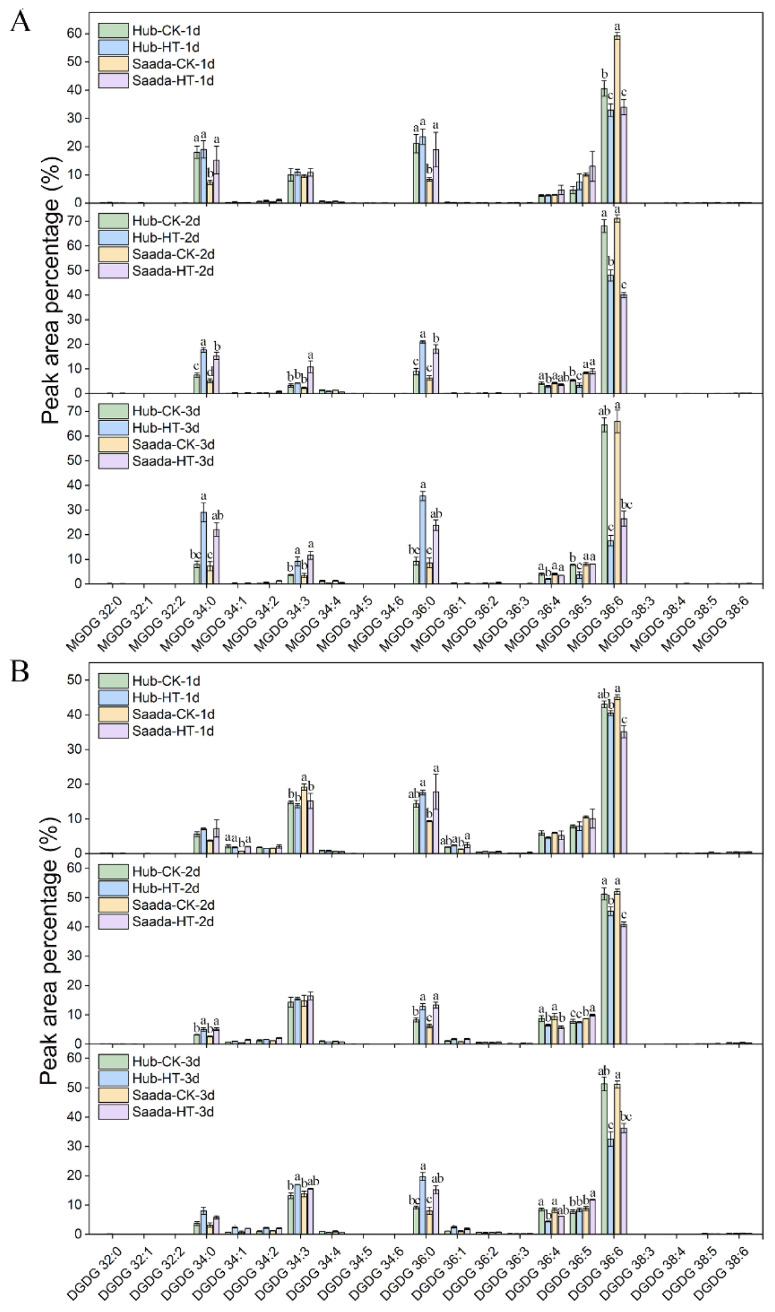
Molecular species composition of MGDG and DGDG in wheat anthers under high-temperature stress. (**A**) Percentage of molecular species in monogalactosyldiacylglycerol (MGDG). (**B**) Percentage of molecular species in digalactosyldiacylglycerol (DGDG). The relative abundance of individual molecular species is shown as a percentage of the total peak area for each lipid class. Anthers were sampled on the first three days (1d–3d) of flowering under normal conditions (CK, 22 °C) and high-temperature (HT, 34 °C) treatment. Data represent mean ± SD from three biological replicates (*n* = 3). Lipid species are denoted on the *X*-axis as total acyl carbons:total double bonds. For each molecular species, different lowercase letters indicate statistically significant temporal changes across days as determined by Fisher’s Least Significant Difference (LSD) test at *p* < 0.05.

The molecular composition of major lipid classes was further examined. In PA, high temperature led to a decrease in PA (36:6), which contains two 18:3 acyl chains, and an increase in PA (34:3) containing 16:0, in both cultivars (Figure 4A). The proportion of polyunsaturated DAG species (18:3/18:3) decreased, while the proportion of DAG (16:0/18:3) increased under high-temperature treatment, (Figure S5). Heat stress consistently reduced the highly unsaturated PC (36:6) in both lines, while elevating PC (34:2), PC (36:4), and PC (36:5) (Figure 4B). A similar trend was observed in PE, marked by a significant rise in PE (34:2) (Figure 4C). Phosphatidylinositol (PI) and phosphatidylserine (PS) showed a decreased unsaturated lipids such as PI (36:6) and PS (36:6), but an increase in 18:2-containing lipids (e.g., PA (34:2), PA (36:5), PI (34:2), PI (36:5)) (Figures S2 and S3). Interestingly, high-temperature treatment induced an increase in PG (36:5) and PG (36:6) but a decrease in PG (34:3), collectively indicating a shift toward higher saturation in PG (Figure 4D). Lysophospholipids (LPC, LPE, LPG) showed similar changes under heat stress. Specifically, levels of polyunsaturated LPC (18:3) and LPE (18:3) decreased, while saturated LPC (16:0) and LPE (18:0) increased (Figure S4). LPG showed only minor alterations, with a notable decrease in LPG (16:0) on the first day of heat treatment. Heat stress also promoted saturation in the galactolipids monogalactosyldiacylglycerol (MGDG) and digalactosyldiacylglycerol (DGDG), evidenced by a reduction in 36:6 and an increase in 34:0 and 36:0 molecular species (Figure 5A,B).

These results suggested dynamic changes in lipid compositions in anthers under high-temperature treatment. Though the proportions of major lipid classes were relatively stable under heat treatment, the composition of lipid acyl chains undergoes notable remodeling. While a general reduction in polyunsaturated fatty acids was observed, certain lipid classes exhibited increased unsaturation, highlighting the distinct functional roles of different lipid types. These findings suggest that lipid-mediated thermotolerance is governed not by changes in individual lipid species alone, but by a coordinated regulation across multiple lipid classes.

### 2.4. Effect of High Temperature on Wax and Cutin Composition

**Figure 6:**
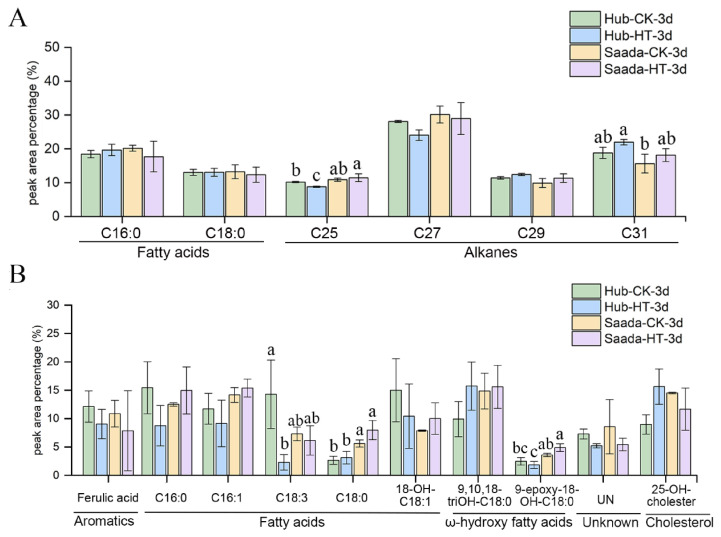
Composition of cuticular lipids in wheat anthers under high-temperature stress. (**A**) Relative content of wax components. (**B**) Relative content of cutin monomers. Anthers were sampled on the first three days (1d–3d) of flowering under normal conditions (CK, 22 °C) and high-temperature (HT, 34 °C) treatment. Data represent the relative abundance (mean ± SD) of each compound class (*n* = 2). For each panel, different lowercase letters indicate significant differences in the relative abundance of specific components between the two cultivars (Hub vs. Saada) as determined by Fisher’s Least Significant Difference (LSD) test at *p* < 0.05.

Although a diverse array of wax and cutin components is known to function in plant abiotic stress responses [52], their contribution to thermotolerance of wheat anthers is still poorly understood. Analysis of wheat anther wax composition revealed that fatty acids and alkanes were the major constituents, including hexadecanoic acid and octadecanoic acid, pentacosane, heptacosane, nonacosane, and hentriacontane. Under high temperature, most wax components showed no significant changes in either the heat-tolerant (Hub) or heat-sensitive (Saada) cultivars on the first and second day of treatment, except for a marked decrease in heptacosane in Saada (Figure S6). On the third day of heat treatment, the heat-tolerant cultivar Hub showed significantly decreased levels of pentacosane and heptacosane but increased levels of nonacosane and hentriacontane (Figure 6A and Figure S6). Such chain length shifts might be related to thermotolerance.

Cutin analysis revealed that hydroxy fatty acids are the predominant components in wheat anthers. After high-temperature exposure, the content of 9,12,15-octadecatrienoic acid decreased significantly in the heat-tolerant cultivar Hub, dropping to only 16.08% of the level observed under normal conditions by the third day of treatment. In contrast, the reduction in this component was not significant in the heat-sensitive cultivar Saada (Figure 6B and Figure S6). Considering that 9,12,15-octadecatrienoic acid is a polyunsaturated cutin component, the differential response suggests that a reduction in unsaturated cutin lipids, leading to an increase in saturated lipid content, may enhance pollen resilience to heat stress.

### 2.5. Effect of High Temperature on Yield-Related Traits

**Figure 7:**
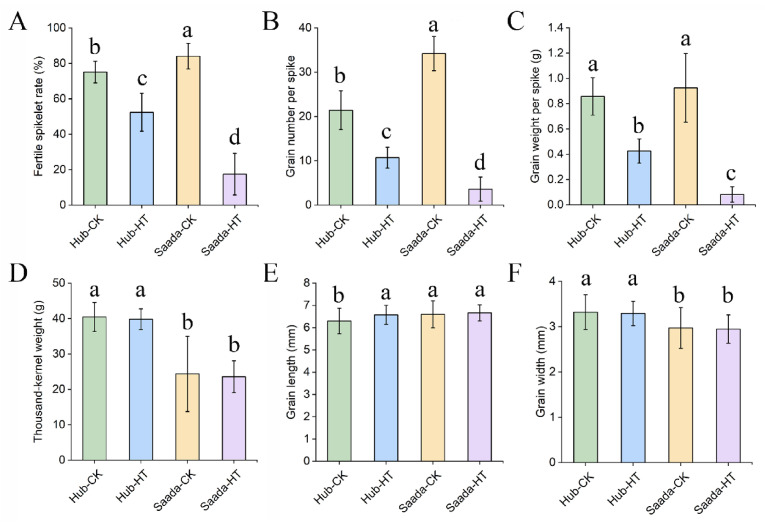
Analysis of yield-related traits after high-temperature treatment during anthesis in wheat cultivars. Yield-related traits including fertile spikelet rate per spike (**A**), grain number per spike (**B**), and grain weight per spike (**C**), thousand kernel weight (**D**), grain length (**E**) and grain width (**F**) were measured at harvest. Plants were exposed to high temperature (34 °C) on the first three days (1d–3d) of flowering and then returned to normal growth conditions until maturity. Data are presented as mean ± SD (*n* = 10). Different lowercase letters indicate statistically significant differences between the control and treatment groups for each cultivar as determined by Fisher’s Least Significant Difference (LSD) test at *p* < 0.05.

Beyond its immediate effects on lipids and pollen fertility, high temperature at anthesis significantly impairs final yield. To evaluate the long-term consequences, plants exposed to heat stress were transferred back to normal growth condition till harvest. Yield-related traits, such as fertile spikelet rate, grain number, grain weight, thousand-grain weight, grain length, and grain width were measured. The results showed significant reductions in the fertile spikelet rate per spike, grain number per spike, and grain weight per spike following heat treatment (Figure 7A–C). Specifically, the fertile spikelet rate in the heat-tolerant cultivar (Hub) decreased from approximately 75.09% to 52.38%, whereas the heat-sensitive cultivar (Saad) dropped from around 84.05% to 17.44%. Similarly, the grain number and grain weight per spike declined by 50.00% and 50.37% in the heat-tolerant line, but by up to 89.47% and 91.18% in the heat-sensitive line. It should be noted that heat treatment has no impacts on the thousand kernel weight of both Hub and Saada (Figure 7D). In contrast, grain length showed significant differences in the heat-tolerant variety Hub, and there was no significant difference in grain width before and after heat treatment (Figure 7E,F). These findings suggest that high-temperature stress during the flowering stage primarily affects reproductive success by impairing pollen fertility, ultimately reducing grain set and yield, particularly in heat-sensitive cultivars.

## 3. Discussion

In the present study, we systematically investigated the physiological and biochemical changes in lipid metabolism in wheat pollen using a heat-resistant line (Hub) and a heat-sensitive line (Saada). Our results revealed a rapid and substantial decline in pollen viability, accompanied by severe surface wrinkling, in the heat-sensitive line under high-temperature stress. In contrast, the heat-resistant line exhibited a more gradual reduction in viability with milder morphological changes. Fatty acid and lipidomic analyses indicated a shift from polyunsaturated to more saturated lipid species under high-temperature conditions. Additionally, wax and cutin analyses demonstrated an increase in very-long-chain alkanes and a reduction in polyunsaturated cutin components, suggesting their potential roles in enhancing heat tolerance.

The detrimental effect of heat stress on cereal pollen fertility is well-established, with its severity depending on genotype and timing. In wheat, high-temperature treatment five days before flowering had the greatest impact on pollen fertility, with a decrease in pollen fertility rate exceeding 60% [18]. Previous studies showed that after 3 days of heat treatment of four wheat materials, the pollen fertility rate of heat-tolerant wheat decreased by 30–40%, while the sensitive materials decreased by around 90% [53]. In other *Poaceae* crops such as rice, after high-temperature treatment, the decrease in pollen fertility rate of heat-tolerant rice varieties was also less than that of heat-sensitive rice varieties [54]. Consistent with these studies, our study demonstrated that high-temperature treatment resulted in decreased fertility in both Hub and Saada on the first day of heat treatment, but the decline was significantly more severe in the heat-sensitive line cultivar. These results indicate that the impact of high-temperature treatment on pollen fertility varies depending on the wheat cultivar. Ultrastructural observations further supported the pollen viability analysis, revealing that high temperatures induce pollen shrinkage and surface collapse, with more severe effects in the heat-sensitive cultivar. The observed reduction in surface granules, may contribute to the loss of fertility, but additional evidence is needed to elucidate the precise relationship between ultrastructural changes and pollen viability under heat stress.

It has been well-established that increased lipid saturation and decreased amount of polyunsaturated fatty acids is associated with heat-tolerance in plants under high temperature [21,55]. We systematically investigated the changes in membrane lipids as well as cuticular lipids in the anthers of two wheat cultivars under high temperature. Consistent with the general trend, total fatty acid analysis showed elevated saturated fatty acids (e.g., C16:0) and reduced polyunsaturates (e.g., C18:3) in both lines. A striking contrast was observed in the heat-sensitive Saada, which uniquely accumulated C18:2. This shift, a reduction in C18:3 and a rise in C18:2, has been observed in many plant species like tomato [55], Arabidopsis [32], and soybean [29], representing a universal strategy of reducing lipid unsaturation to alleviate heat-induced damage. Lipidomics analysis further revealed an increase in 18:2-containing lipid species, such as 34:2, 36:4, and 36:5 in PC and PE, suggesting that the observed changes in fatty acid composition are likely the result of adaptive adjustments in lipid metabolism under high-temperature stress.

Lipid changes at different temperatures in plants are not only observed in fatty acid composition but also in the proportion of glycerolipid species. A considerable number of studies have focused on the lipid changes in leaf tissues under temperature stress [33,56,57,58,59,60,61]. However, due to tissue-specific metabolic pathways, lipid compositional changes in anther and pollen have been poorly understood. Our lipidomics analysis uncovered a heat-induced increase in PA and LPG, with other classes showing cultivar-specific shifts; e.g., PC and PE decreased in the tolerant Hub, while MGDG was significantly reduced in the sensitive Saada. Transcriptome studies have identified thousands of differentially expressed genes in wheat anthers under heat stress [54]. Consistent with the increased PA levels, a gene encoding phospholipase D alpha (PLD), which catalyzes the production of PA from phospholipids, was up-regulated in wheat anthers in response to heat. In addition, the concurrent rise in PA and decline in these phospholipids in Hub points to a heat-induced bottleneck in the phospholipid biosynthesis pathway [62]. MGDG is believed to play a crucial role in regulating fatty acid desaturation within plastids, as the desaturation rate of fatty acids in MGDG is significantly higher across various temperatures [63]. Transcriptome studies also showed monogalactosyldiacylglycerol synthase (MGDS) encoding genes were repressed in wheat anthers under heat stress [61]. The significant reduction of MGDG in Saada may be associated with its lower tolerance to heat stress.

While the relative abundance of major lipid classes remained largely stable under heat stress, analysis revealed substantial reorganization of acyl chain compositions. A consistent trend toward increased saturation was evident from the elevated proportions of saturated lipid species (e.g., PC (34:2), PE (34:2), DGDG (36:0)) and the concomitant decline in polyunsaturated species (e.g., PA (36:6), PC (36:6), MGDG (36:6)) [62]. This reprogramming, which likely stabilizes pollen membranes under thermal stress, aligns with the reported increased flux of 16:0-containing DAG into plastids under high temperature [33], as reflected by the accumulation of DAG (16:0/18:2) and MGDG (34:0) in our data. These results suggested that wheat anthers respond to thermal stress by modulating lipid metabolism to reduce membrane lipid unsaturation, thereby enhancing membrane stability and pollen viability. Unlike the consistently increased polyunsaturated lipids in leaves [33,64], this study revealed a decrease in the proportion of PG (34:3), while PG species with higher unsaturation levels such as PG (36:4), PG (36:5) and PG (36:6) increased. These results suggested an increased biosynthesis of PG in the ER, while the plastidial pathway might be repressed. These counter intuitive changes in the polyunsaturated lipids implied that different lipid classes may be governed by distinct regulatory mechanisms, and that pollen adaptation to heat stress involves the coordinated remodeling of multiple lipid species rather than reliance on a single key lipid.

In addition to membrane lipids, studies have found that the wax content in wheat leaves significantly increases under high-temperature stress [65,66,67]. However, the changes in cuticular lipids during anther development remain unexplored in wheat. Our study fills this knowledge gap by demonstrating a cultivar-specific accumulation of very-long-chain alkanes—a response observed only in the heat-tolerant line, implying a functional contribution to thermotolerance. This aligns with the established role of alkanes in plant drought adaptation [68,69]. Concurrently, we found that cutin unsaturation decreased, as evidenced by the reduction of octadecatrienoic acid (18:3). This shift toward greater cuticular saturation likely works in concert with alkane accumulation to enhance pollen heat resilience.

Our study showed that heat-sensitive cultivar (Saad) experienced a more drastic reduction in fertile spikelet rate, grain number per spike and grain weight per spike after high-temperature treatment in comparison to Hub. In contrast, no significant differences were observed in spike length, thousand-grain weight, grain width, and grain length between the two cultivars. This outcome likely reflects the timing of heat stress application, which in this study heat stress was imposed only for three days at anthesis, a period known to critically impact pollen viability and seed setting [70]. This selective impact directly links the preserved yield performance of tolerant lines to their ability to maintain pollen fertility and successful fertilization under heat stress.

## 4. Materials and Methods

### 4.1. Plant Materials and Growth Conditions

Two wheat lines with contrasting thermotolerance were selected for this study: the heat-tolerant Hub and the heat-sensitive Saada [50]. Both lines were cultivated under identical controlled conditions (temperature, light, and moisture) until the heading stage. When anthers extrude from the florets, plants designated for heat stress were transferred to a growth chamber set at 34 °C (day) and 28 °C (night). Anthers and pollen were collected on the first, second, and third day of heat treatment, respectively, between 2:00 P.M. and 4:00 P.M. During sampling, anthers were collected with forceps, immediately frozen in liquid nitrogen in 2 mL centrifuge tubes, and stored at −80 °C for subsequent analysis.

### 4.2. Pollen Fertility Assessment and Structural Observation

To ensure sampling at the same development stage, anthers were collected from the four side florets of the middle segment of wheat spikes using clean forceps and placed on a microscope slide. A drop of distilled water was added, and the anthers were gently crushed to release the pollen grains. Subsequently, 1–2 drops of iodine-potassium iodide solution were applied, and the sample was observed under an optical microscope at 10× magnification. Pollen grains that were plump, uniformly dark blue-stained, and spherical were classified as fertile, whereas those that were irregularly shaped, unstained, or unevenly stained were considered sterile.

Pollen ultrastructure analysis was conducted using scanning electron microscopy (SEM) at the Electron Microscopy Platform of Huazhong Agricultural University. At 10–11 a.m. during the flowering period, approximately 20 fresh anthers from both the control group and the high-temperature treatment group were placed in 2.5% glutaraldehyde fixative. Subsequently, vacuum fixation was carried out, and then gold powder was sprayed on the surface of the sample. The SEM (JSM-6390/LV, JEOL, Tokyo, Japan) device for observing crystal morphology by field emission uses an acceleration voltage of 10 kV and a working distance of 8.4 mm.

### 4.3. Fatty Acid Extraction and Analysis

The fatty acid methylation extraction method was employed. Glass tubes were rinsed with chloroform to remove any potential lipids present. Samples were then added to the glass tubes. Next, 1 mL of a methanol solution containing 5% H_2_SO_4_ was added, followed by 20 μL of a methanol solution containing 0.2% butylated hydroxytoluene. The glass tubes were then placed in an 80 °C water bath for 2 h. After the samples cooled to room temperature, 1 mL of 0.9% (*w*/*v*) NaCl and 1 mL of n-hexane were added. The tubes were vortexed vigorously and centrifuged at 2000 r/min for 5 min. Following centrifugation, the upper organic phase was transferred to a new tube, and the solvent was evaporated under nitrogen gas before being redissolved in n-hexane. The newly obtained n-hexane containing the fatty acids was transferred to a specially designed vial for analysis.

Analysis was performed using GC-FID (8890 GC-FID, Agilent, Santa Clara, CA, USA). The temperature program used was: hold at 160 °C for 2 min, then increase to 240 °C and run for 19 min. The retention times of the various fatty acids were determined using standards and GC-MS. After sample analysis, the GC software Qualitative Analysis 10.0 was used to extract the peak areas of the fatty acids and calculate the relative molar fractions of each fatty acid component. Three biological replicates were performed for each sample.

### 4.4. Lipid Extraction and Analysis

Twenty fresh anthers (1.9–2.7 mg dry weight per sample) with three biological replicates per treatment were processed. Samples were added to glass tubes, followed by the addition of 1 mL of isopropanol containing 0.01% BHT heated to 75 °C, and then placed in a 75 °C water bath for 15 min. Subsequently, 3 mL of a chloroform:methanol:water mixture (30:41.5:3.5) was added, and the samples were subjected to ultrasonic oscillation for 1 h. During this period, the water bath was refreshed every half hour to prevent the temperature from exceeding 40 °C. After slight centrifugation at 1000 r/min for 2 min, the solution was transferred to a new glass tube. The ultrasonication step was repeated until the plant tissue turned white. Then, 2 mL of 1 M KCl solution was added to the extract, followed by vortex mixing and centrifugation to remove the aqueous layer. This was followed by the addition of 2 mL of distilled water, another round of vortex mixing and centrifugation, and removal of the water layer. The extract was dried under nitrogen and redissolved in chloroform, then transferred to a sample vial. All samples were redissolved to achieve a standardized lipid concentration of 10 mg dry weight (DW) per mL in chloroform prior to LC-MS analysis.

Analysis was conducted using a QTRAP 4000 (ABSciex, Framingham, MA, USA) mass spectrometer coupled with an LC20A HPLC system (Shimadzu, Kyoto, Japan). Detection was performed in positive ion mode. The lipids were separated on an Accucore C30 column (100 × 2.1 mm, 2.6 μm particle size, Waters) using two eluents, A and B. Eluent A was composed of water:methanol:acetonitrile:300 mM ammonium acetate in a 20:20:20:1 ratio (*v*/*v*/*v*/*v*), and eluent B consisted of isopropanol:methanol:300 mM ammonium acetate in a 180:20:3 ratio (*v*/*v*/*v*). The gradient elution program was set as follows: from 25% to 40% B over 1–2 min; from 40% to 95% B over 2–4 min; held at 95% B from 4–18 min at a flow rate of 0.3 mL/min, with a sample injection volume of 2 μL. After the run, chromatographic peak areas were measured for relative quantification using MultiQuant software SCIEX OS 3.3.1.43 (ABSciex, USA). The total ion chromatogram of the lipidome is provided in Supplementary Materials (Figure S7A). Three biological replicates were performed for each sample.

After LC-MS data acquisition, the peak area of each lipid was extracted as raw intensity. For total lipids, normalization was performed based on the total peak area signals of lipid species with different head groups to represent the relative content of each head group-specific lipid. By comparing the ratios of lipid relative content across heat treatment, changes in the relative abundance of head group-specific lipids in total lipids under heat stress were analyzed. For lipids within the same head group, normalization was performed using peak area signals of different acyl chains to represent the relative content of each acyl chain-specific lipid. By comparing changes in the relative content of acyl chains within the same head group, metabolic alterations in lipid acyl chains under heat stress were examined. The relative lipid content was calculated as the ratio of peak area signals to total lipid signals (or signals within the same head group), defined as peak area percentage. The mean and standard deviation of peak area percentages for each treatment were calculated.

### 4.5. Extraction and Analysis of Wax and Cutin

Before extracting the wax, the anther samples were weighed and recorded. Samples were placed into glass tubes, to which 2 mL of chloroform containing 10 μg of tetracosane was added. The mixture was shaken at room temperature for 30 s. The chloroform was then transferred to another glass tube, dried under nitrogen, redissolved in 0.5 mL of chloroform, and transferred to a sample vial. Prior to GC-MS/FID detection, 40 μL of BSTFA reagent (Sigma-Aldrich, St. Louis, MO, USA, Cat. 15238) was added to the sample, which was then maintained at 70 °C for 1 h for derivatization.

For the extraction of cutin, samples were immersed in 3 mL of a methanol:chloroform mixture (1:1, *v*/*v*) and stirred continuously at room temperature at 200 r/min. The solvent was changed every 4 h, with a total of three changes, after which the sample was dried under nitrogen and further dried in an oven at 60 °C for at least 12 h. Then, 3 mL reaction medium composed of methanol/concentrated sulfuric acid/chloroform (10:0.5:1, *v*/*v*/*v*) containing 10 μg of methyl heptadecanoate and 5% butylated hydroxytoluene (BHT, the final concentration was 0.01%) was added, and the mixture was heated in an 80 °C water bath for 2 h. After cooling to room temperature, 1.5 mL of 0.9% NaCl was added to terminate the reaction. Then, the sample was extracted three times with 2 mL of hexane and chloroform (4:1, *v*/*v*), each time vortexing at maximum speed for 30 s and optionally centrifuging for 2 min. To each of the collected organic layers, 1 mL of 0.9% NaCl (*w*/*v*) was added and vortexed for 30 s. The upper organic layer was collected, dried under nitrogen, redissolved in 0.5 mL of chloroform, and transferred to a sample vial. Prior to GC-MS/FID detection, 40 μL of BSTFA was added and the sample was maintained at 70 °C for 1 h for derivatization.

Waxes and cutin monomers were analyzed by GC-MS/FID (GCMS-8890-5977B, Agilent, USA) equipped with Hp-5ms-UI column (Agilent, USA). Briefly, the column was operated with the helium carrier gas (>99.999% purity) at a flow rate 1 mL/min and splitless injection at 280 °C. For wax detection, set the oven temperature at 50 °C for 2 min, increase by 20 °C/min to 250 °C, followed by 5 °C/min to 300 °C for 4 min and 10 °C/min to 320 °C for 2 min. For cutin detection, set the oven temperature at 50 °C for 2 min, increase by 50 °C/min to 180 °C, followed by 4 °C/min to 270 °C and 12 °C/min to 320 °C for 6 min. The ion source was operated in electron impact (EI) mode at 70 eV. Compound identification is carried out using the full scanning mode (*m*/*z* range of 50–650). The conditions of the FID detector were as follows: temperature 280 °C, hydrogen flow rate 30 mL/min, and air flow rate 300 mL/min. Finally, Mass spectral feature ions of wax and cutin were identified using full scan mode and quantification analysis was performed using the FID mode. The chromatograms are provided in Supplementary Materials (Figure S7B,C). Two biological replicates were performed for each sample.

### 4.6. Agronomic Trait Investigation

After high-temperature treatment, plants were returned to normal conditions and cultivated alongside untreated controls until seed maturity. At maturity, agronomic traits were measured, including panicle length, number of fertile and sterile spikelets per panicle, grain number per panicle, grain weight per panicle, and thousand-grain weight.

## 5. Conclusions

This study establishes that lipid metabolic remodeling is a key component of wheat anther adaptation to high-temperature stress. Through integrated physiological and lipidomic analyses of contrasting cultivars, we found that heat stress severely compromises pollen fertility, with the extent of damage being genotype-dependent. Importantly, we observed that thermal adaptation is associated with a marked shift in lipid composition—including increased saturation of fatty acids, reorganization of membrane lipids, and specific changes in cuticular alkane and cutin profiles. These biochemical adjustments were further linked to yield outcomes, as heat stress during flowering led to greater yield loss in sensitive cultivars due to reduced grain set. Our findings support the proposition that lipid-based pathways are integral to thermotolerance, offering both new perspectives for understanding heat adaptation and a theoretical basis for developing climate-resilient wheat.

## Figures and Tables

**Table 1:** Double bond index (DBI) of fatty acids in wheat anthers after high-temperature treatment.

Wheat Materials	Control	High Temperature
Saada	1.94 ± 0.03 a	1.58 ± 0.04 b
Hub	1.93 ± 0.06 a	1.42 ± 0.02 c

Anthers were sampled on the third day of high-temperature treatment (HT, 34 °C) and control (CK, 22 °C). DBI was calculated based on fatty acid composition of anthers. Different letters indicate significant difference (*p*-value < 0.05).

## Data Availability

Data will be made available on request.

## Supplementary Materials

The following supporting information can be downloaded at: https://www.mdpi.com/article/10.3390/ijms262311426/s1.
