# Supplementary material for: Biochemical Insights into Lipid Remodeling in Wheat Anthers Under High-Temperature Stress
Source: Int J Mol Sci. 2025 Nov 26;26(23):11426. doi: 10.3390/ijms262311426 (PMC12691984; doi:10.3390/ijms262311426)
Supplement: Supplementary file 1 [file ijms-26-11426-s001.zip › ijms-3651981-supplementary.pdf]

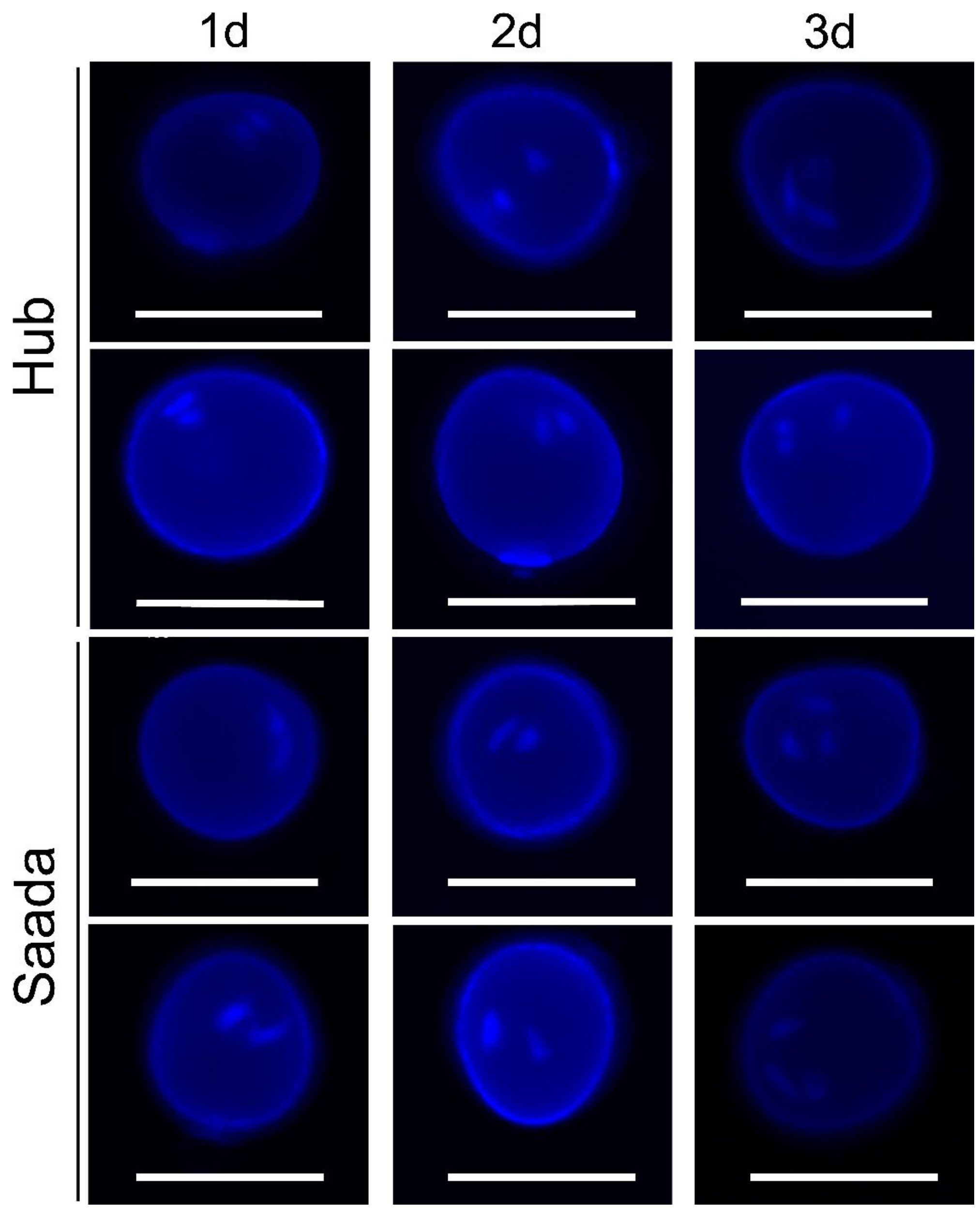

Figure S1. DAPI staining confirms synchronized pollen development. Pollen from both Hub and Saada progressed from the binucleate stage (1-2 d) to the trinucleate stage (3 d) during sampling, as shown by DAPI staining. Scale bars: 50 μm.

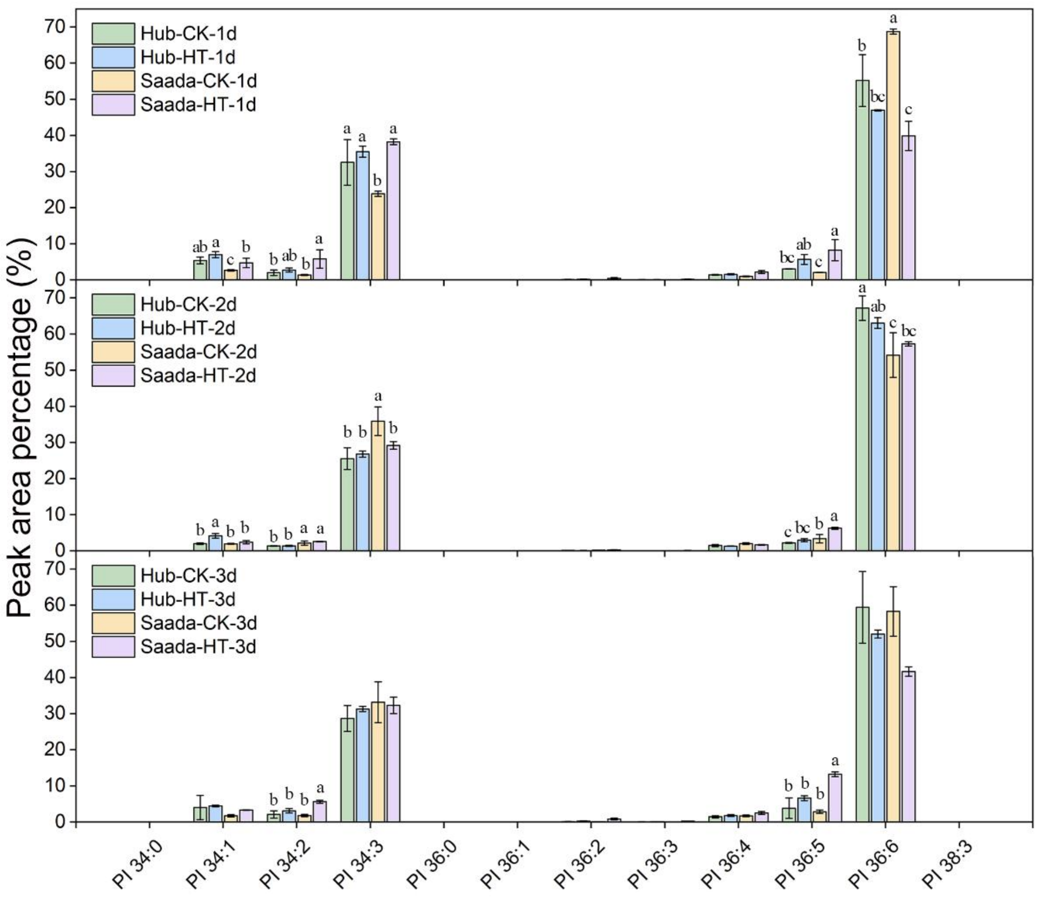

Figure.S2 Dynamic changes in phosphatidylinositol (PI) composition under heat stress. The profile shows the shift in relative abundance of specific PI species over the first three days of flowering under continuous high temperature (34°C). Molecular species are identified by their acyl chain composition (total carbons:double bonds) on the X-axis. Statistical analysis (LSD test, $P < 0.05$) reveals significant temporal progression for numerous species, as indicated by different lowercase letters, highlighting the active reorganization of this lipid class under stress. Data are presented as mean ± SD ($n = 3$).

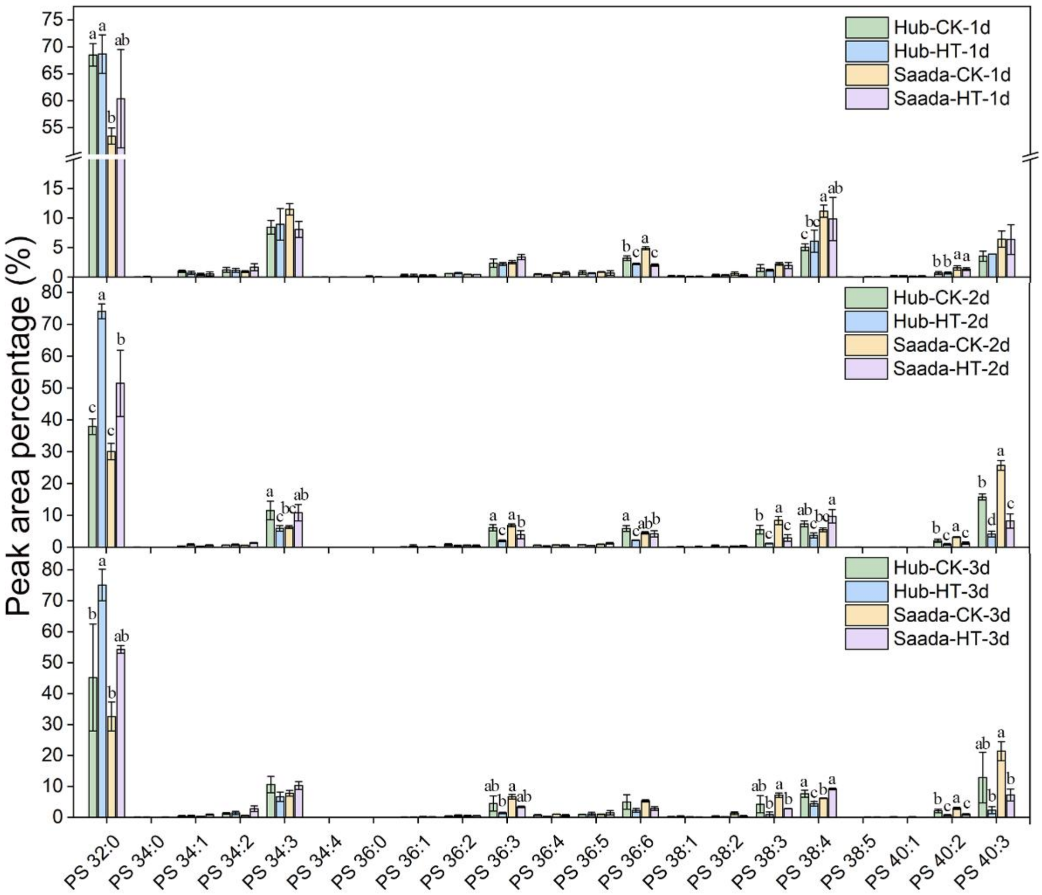

Figure.S3 Dynamic changes in phosphatidylserine (PS) composition under heat stress. Relative abundance of PS molecular species in pollen during the first three days of high-temperature treatment (34°C). The X-axis indicates lipid species (total acyl carbons:total double bonds). Different lowercase letters for each species signify significant temporal changes across days (LSD test, $P < 0.05$). Data are presented as mean ± SD ($n = 3$).

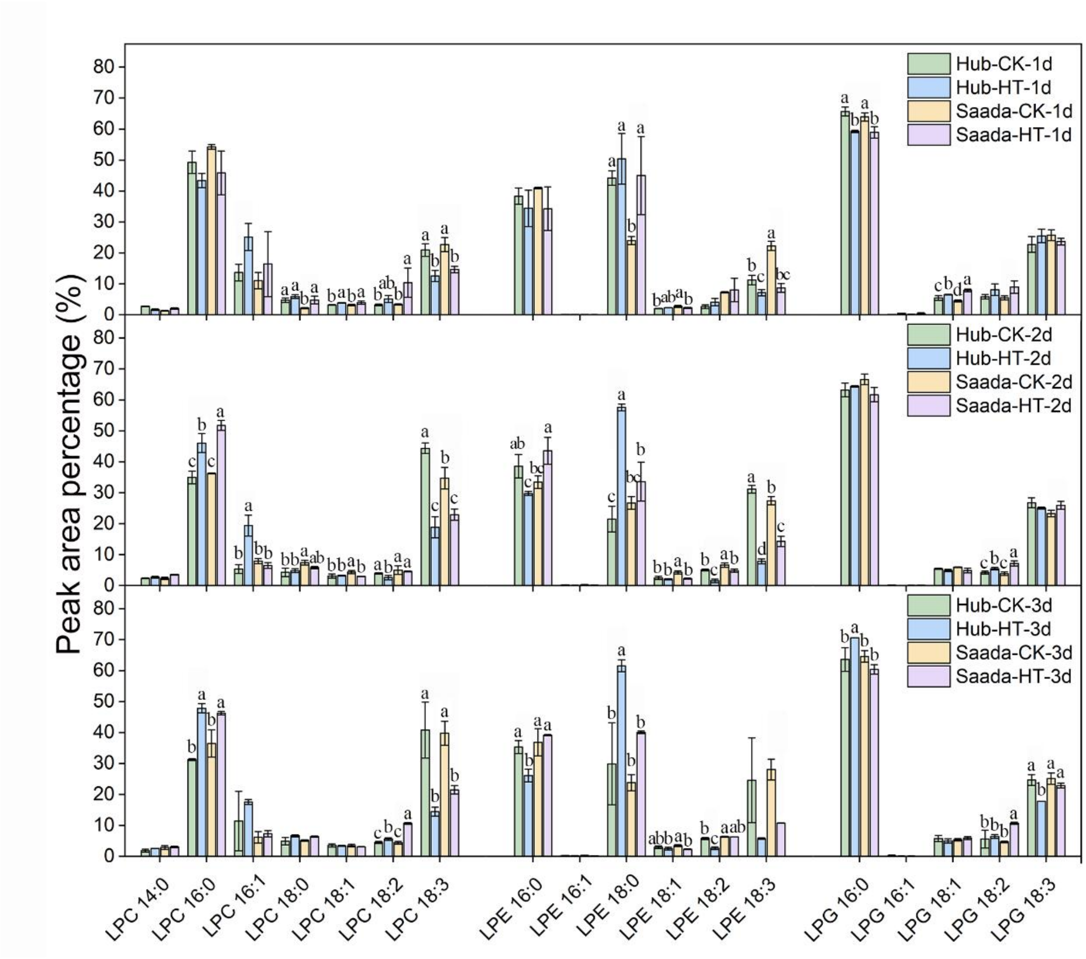

Figure.S4 Dynamic changes in lysophospholipid composition under heat stress. Relative abundance of molecular species for LPC, LPE, and LPG in pollen during the first three days of high-temperature treatment (34°C). The X-axis indicates lipid species (total acyl carbons:total double bonds). Different lowercase letters for each species signify significant temporal changes across days (LSD test, $P < 0.05$). Data are presented as mean ± SD ($n = 3$).

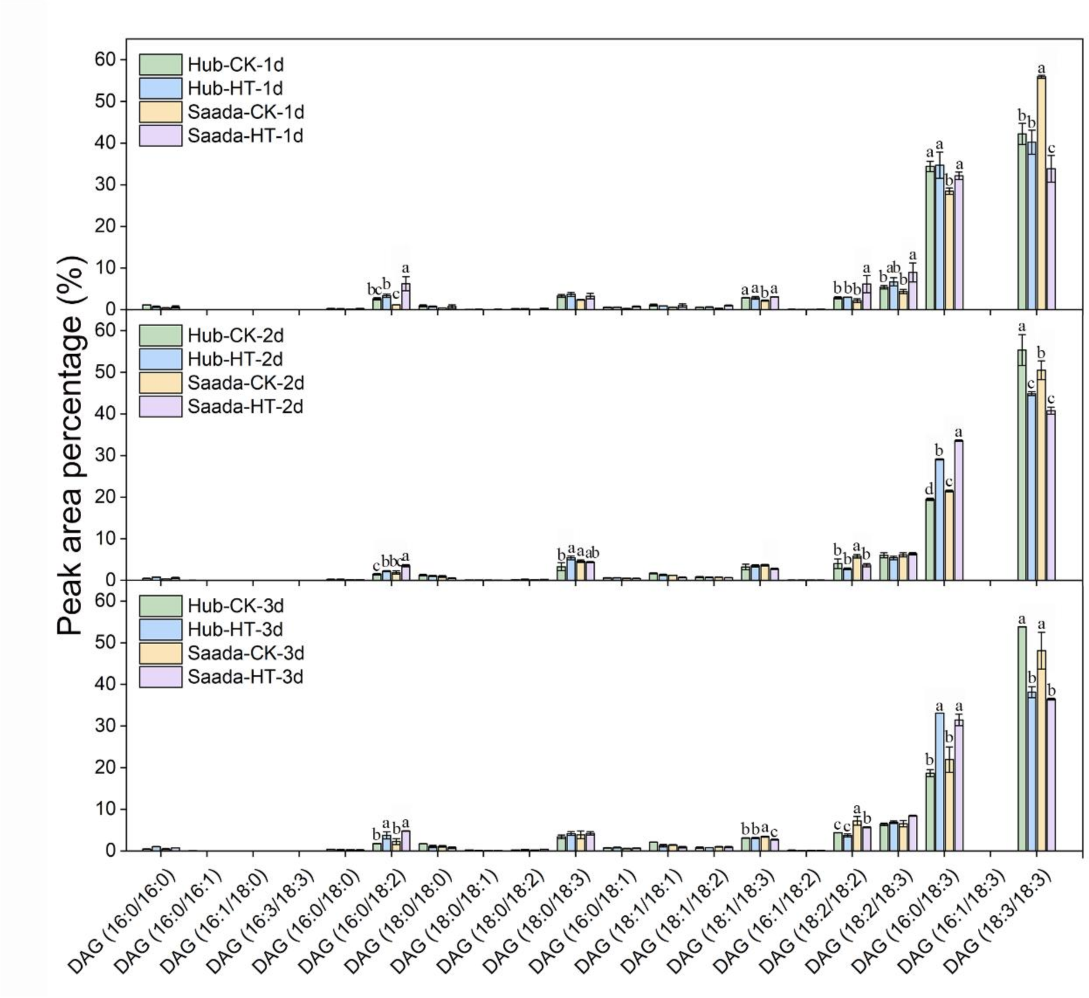

Figure.S5 Dynamic changes in diacylglycerol (DAG) composition under heat stress. Relative abundance of DAG molecular species in anthers during the first three days of high-temperature treatment (34°C). The X-axis indicates lipid species (total acyl carbons:total double bonds). Different lowercase letters for each species signify significant temporal changes across days (LSD test, *P*< 0.05). Data are presented as mean ± SD (*n* = 3).

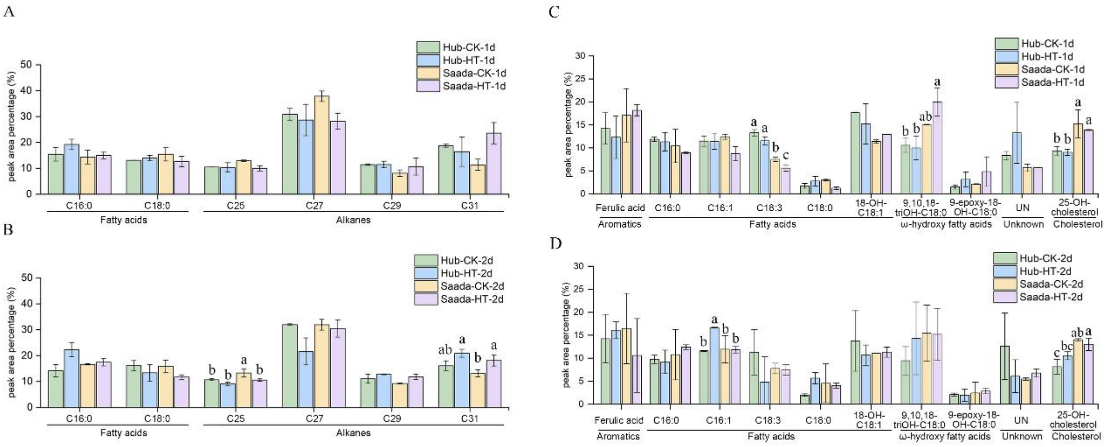

Figure.S6 Composition of cuticular lipids in wheat anthers after two days of high-temperature stress. Means with different letters are significantly different according to the least significant difference (LSD) test at $P<0.05$.

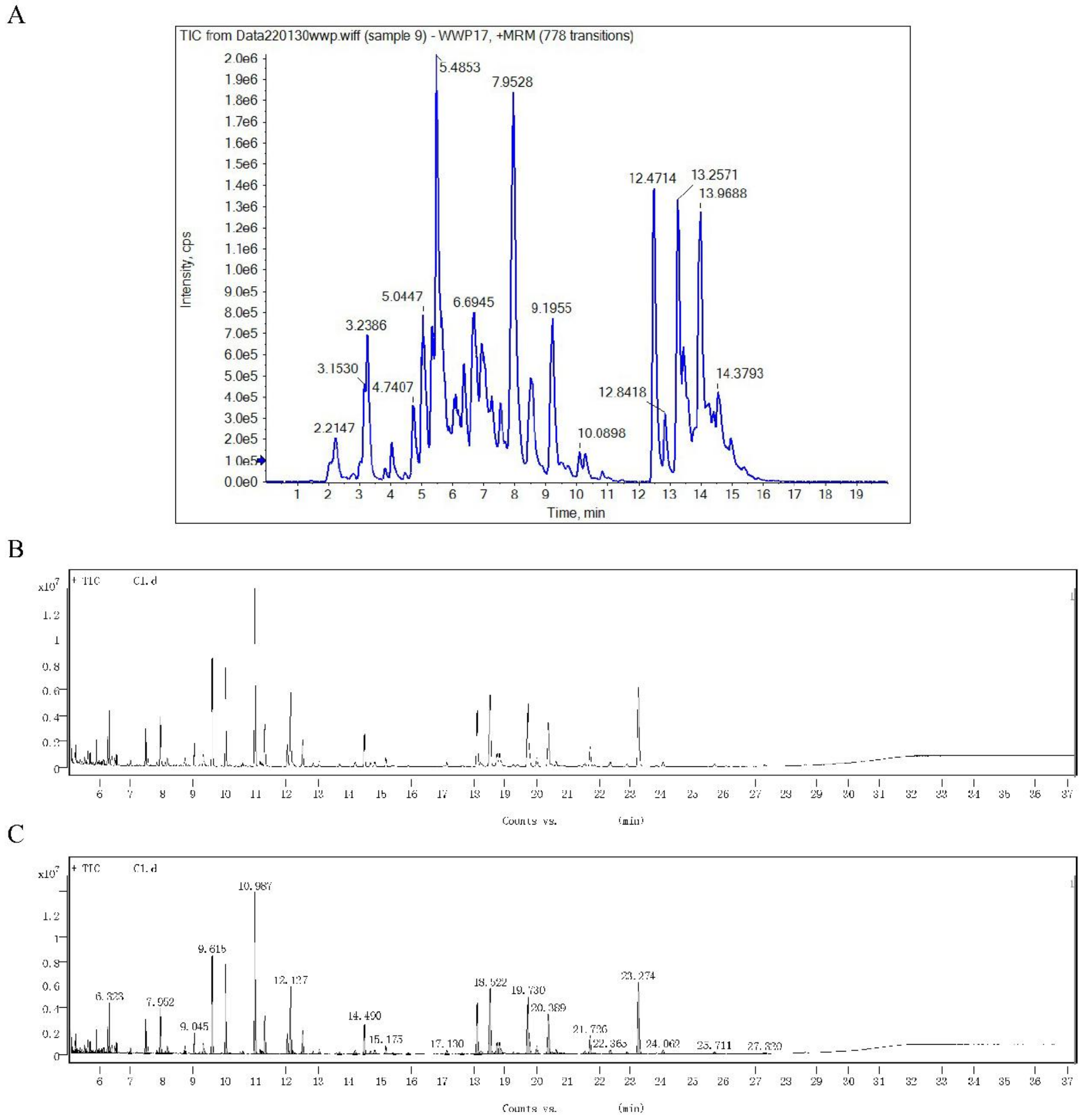

Figure S7. Representative chromatograms for lipidomic and cuticular lipid analyses. A: Chromatographic profiles of the lipidome. B-C: Corresponding chromatograms for cuticular waxes and cutin.
